# When and Why Does Subjective Age Boost Competence and Proactive Work Behavior?

**DOI:** 10.1093/geroni/igaa057.2110

**Published:** 2020-12-16

**Authors:** David Weiss

**Affiliations:** Leipzig University, Leipzig, Sachsen, Germany

## Abstract

Subjective age bias suggests that middle-aged and older adults feel relative younger, whereas adolescents and young adults often feel older than their chronological age. However, we still know very little about its social conditions and consequences across the life span particularly within the work domain. Across three studies (correlational, experimental, and field: Ns = 650, 16-85 years), we show that feeling older (among younger adults) and younger (among older adults) is triggered by undesirable age stereotypes concerning competence and status of young and later adulthood and desirable age stereotypes of midlife. We further demonstrate that feeling relatively older among young adults and younger among older adults increases individuals’ self-perceived competence at work and predicts proactive behavior such as speaking up. We discuss subjective age bias as socially-mediated phenomenon and how it affects behavior at work across the life span.

